# Priming with Toll-like receptor 3 agonist or interferon-gamma enhances the therapeutic effects of human mesenchymal stem cells in a murine model of atopic dermatitis

**DOI:** 10.1186/s13287-019-1164-6

**Published:** 2019-02-22

**Authors:** Arum Park, Hyojung Park, Jisun Yoon, Dayeon Kang, Myoung-Hee Kang, Y-Y Park, Nayoung Suh, Jinho Yu

**Affiliations:** 1 Asan Institute for Life Sciences, Asan Medical Center, Seoul, 05505 South Korea; 2 Department of Pediatrics, Mediplex Sejong Hospital, Incheon, 21080 South Korea; 3 Department of Pharmaceutical Engineering, College of Medical Sciences, Soon Chun Hyang University, Asan, 31538 South Korea; 4 Department of Convergence Medicine, Ulsan College of Medicine, Seoul, 05505 South Korea; 5 Department of Pediatrics, Asan Medical Center, University of Ulsan College of Medicine, Seoul, 05505 South Korea

**Keywords:** Mesenchymal stem cells, Priming, Atopic dermatitis, Toll-like receptor 3 agonist, Interferon-γ

## Abstract

**Background:**

Atopic dermatitis (AD) is a chronic and relapsing inflammatory skin disease. Great efforts have been recently made to treat AD using mesenchymal stem cells (MSCs), which have immunomodulatory functions. However, the immunomodulatory effects of MSCs need to be enhanced for clinical application in the treatment of AD.

**Objectives:**

To evaluate and characterise the therapeutic effects of human Wharton’s jelly-derived MSCs (WJ-MSCs) primed with the Toll-like receptor 3 agonist poly I:C or interferon-γ (IFN-γ) in a murine model of AD.

**Methods:**

Mice were treated with *Aspergillus fumigatus* extract to induce AD and then subcutaneously injected with non-primed, poly I:C-primed or IFN-γ-primed WJ-MSCs. Clinical symptom scores, transepidermal water loss (TEWL), histological characteristics and cytokine levels were determined. Transcriptome profiling and pathway analyses of primed WJ-MSCs were conducted.

**Results:**

The clinical symptom score and TEWL in skin lesions were reduced in mice administered non-primed and primed WJ-MSCs. Epidermal thickness and inflammatory cell infiltration in skin lesions were reduced more in mice administered primed WJ-MSCs than in mice administered non-primed WJ-MSCs. Secretion of interleukin-17 was significantly reduced in skin draining lymph nodes of mice administered primed WJ-MSCs. Genomics and bioinformatics analyses demonstrated the enrichment of certain pathways specifically in WJ-MSCs primed with poly I:C or IFN-γ.

**Conclusions:**

Priming with poly I:C- or IFN-γ improved the therapeutic effects of WJ-MSCs in a murine model of AD. This study suggests that priming with poly I:C or IFN-γ enhances the immunomodulatory functions of WJ-MSCs and can be used as a novel therapeutic approach for AD.

**Electronic supplementary material:**

The online version of this article (10.1186/s13287-019-1164-6) contains supplementary material, which is available to authorized users.

## Background

Atopic dermatitis (AD) is a chronic and relapsing inflammatory skin disease characterised by pruritic skin lesions, dysregulated immune responses and disrupted epidermal barrier function [1]. AD first appears during childhood in most cases, but also develops in a substantial number of adults. Approximately 20% of children and 1–3% of adults with AD have moderate-to-severe disease [2]. The pathogenesis of AD is typically characterised by increased T helper (Th) 2-mediated inflammatory responses, including release of IgE, recruitment of eosinophils and production of interleukin (IL)-4, IL-5 and IL-13. Th17 cells also participate in this process by producing IL-17 [2–5]. AD is treated with systemic and topical anti-inflammatory, anti-pruritic and immunosuppressive agents. However, current treatments are focused on reducing the severity and frequency of symptoms. Therefore, a new method must be developed to effectively treat AD.

Mesenchymal stem cells (MSCs) are multipotent and non-haematopoietic stem cells that can differentiate into various cell types and have immunomodulatory functions [6–8]. MSCs elicit potent immunosuppressive and anti-inflammatory effects by interacting with lymphocytes associated with the innate and adaptive immune systems [9]. The immunomodulatory effects of MSCs are dependent on the inflammatory environment. Recent studies demonstrated that Toll-like receptor (TLR) activation modulates the functions and responses of MSCs by changing the inflammatory microenvironment. For example, treatment with a TLR4 agonist induces pro-inflammatory responses (increased levels of IL-6, IL-8 and transforming growth factor-β), while treatment with a TLR3 agonist promotes immunosuppressive responses (increased levels of IL-10, indoleamine-2,3-dioxygenase and prostaglandin E_2_) in MSCs [10, 11]. Interferon-gamma (IFN-γ) is a pro-inflammatory cytokine involved in transcriptional regulation of immunologically relevant genes, including major histocompatibility complex and the co-stimulatory molecules cluster of differentiation (CD) 80 and CD86. Sheng and Krampera reported that IFN-γ-treated MSCs exhibit immunosuppressive functions in vitro [8, 12]. In a recent clinical trial, ~ 55% of AD patients exhibited improved symptoms following treatment with a high dose of umbilical cord blood-derived MSCs [13]. Strategies must be developed to enhance the therapeutic effects of MSCs.

The current study investigated the therapeutic effects of human Wharton’s jelly-derived MSCs (WJ-MSCs) primed with poly I:C or IFN-γ in mice with *Aspergillus fumigatus* (*Af*)-induced AD. We further investigated how these pro-inflammatory molecules affect the functions of WJ-MSCs by performing transcriptome profiling and pathway analyses.

## Materials and methods

### Isolation, culture and priming of WJ-MSCs

A fresh human umbilical cord was obtained following the birth of a baby at full-term (Asan Medical Center IRB No.: 2015-0303). The umbilical cord was rinsed in normal saline, and arteries, veins and adventitia were removed. Thereafter, the tissue was cut into small pieces (2–3 mm) and digested in Dulbecco’s modified Eagle’s medium (DMEM; Invitrogen-Gibco, NY, USA) containing 0.1% collagenase A (Roche, Mannheim Germany) for 3 h at 37 °C in a shaking incubator. Cells were filtered and then cultured in DMEM supplemented with 10% fetal bovine serum, 50 μg/mL penicillin and 100 μg/mL streptomycin (Invitrogen-Gibco). WJ-MSCs were grown to 60–70% confluency in growth medium prior to priming with poly I:C or IFN-γ. For priming with poly I:C, WJ-MSCs were treated with 1 μg/mL poly I:C for 1 h, washed with fresh medium and cultured for an additional 48 h. Alternatively, WJ-MSCs were treated with 10 ng/mL IFN-γ for 24 h.

### Murine model of AD and administration of WJ-MSCs

Eight-week-old BALB/c female mice were purchased from Orient Bio Inc. (Seongnam, South Korea) and kept in a specific pathogen-free environment. All aspects of animal care and treatment were approved by the Institutional Animal Care and Use Committee of Asan Medical Center and Ulsan University College of Medicine (2015-02-198). AD was induced by applying 40 μg of *Af* extract (Greer Laboratories, NC, USA) to the shaved dorsal skin of mice twice with an interval of 2 weeks. Mice were subcutaneously injected with WJ-MSCs on day 24 and sacrificed on day 29 for further analyses.

### Clinical scoring and assessment of epidermal permeability barrier function

Dorsal skin lesions were clinically scored by a single investigator prior to sacrifice. Dryness, scaling, erosion, haemorrhage and excoriation were scored as 0 (absent), 1 (mild), 2 (moderate) and 3 (severe). These individual scores were summed to calculate the clinical symptom score. Epidermal permeability barrier function was evaluated by measuring transepidermal water loss (TEWL) using a Vapometer® SWL-3 instrument (Delfin Technologies Ltd., Kuopio, Finland) on the same day as clinical scoring.

### Histological examination of mouse skin

Skin samples were fixed with 10% formalin, embedded in paraffin, cut into sections (5 μm thick) and stained with haematoxylin-eosin and toluidine blue. The mean numbers of eosinophils, neutrophils, lymphocytes and mast cells in eight random fields per slide (magnification, × 400) were calculated. Epidermal and dermal thickness was measured using IMT i-Solution software.

### Measurement of cytokine levels

Skin draining lymph nodes (LNs; inguinal, axillary, brachial and superficial cervical) were harvested from mice without surrounding fat and dissociated using a 40-μm cell strainer (SPL Life Sciences, Pocheon, Korea). LN cells were seeded into a 24-well culture plate at a density of 2 × 10^6^ cells/well and treated with anti-CD3 (3 μg/mL) and anti-CD28 (1 μg/mL) antibodies to stimulate and expand T cells. After 2 days, the medium was harvested and levels of IL-10, IL-13, IFN-γ and IL-17A were determined using enzyme-linked immunosorbent assay (ELISA) kits (eBioscience, CA, USA).

### In vivo imaging

WJ-MSCs were labelled with Qtracker® 800 (Invitrogen, CA, USA) according to the manufacturer’s protocol and subcutaneously injected into mice. Labelled cells were assessed 24, 48, 72 and 120 h after injection using an IVIS Spectrum In Vivo Imaging System (PerkinElmer, MA, USA). Imaging data were analysed using Optix MX3 software (Advanced Research Technologies Inc., QC, Canada).

### Microarray and pathway analyses

Total RNA was isolated from non-primed (control), poly I:C-primed and IFN-γ-primed WJ-MSCs using a mirVana Isolation Kit (Thermo Fisher Scientific, MA, USA). Extracted total RNA (500 ng) was used for labelling and hybridisation to a Human BeadChip V4 microarray (Illumina, CA, USA) in accordance with the manufacturer’s protocols. The chips were scanned using an Illumina BeadArray Reader. Thereafter, the microarray data were normalised using the quantile normalisation method of the Linear Models for Microarray Data package in the R language environment. The expression level of each gene was log2-transformed prior to further analyses. Canonical pathway, functional network and comparison analyses were conducted using Ingenuity Pathway Analysis (IPA) software (Qiagen, Hilden, Germany).

### Statistical analysis

All groups were compared using Student’s *t* test. Statistical analyses were performed using SAS software, version 9.4 (SAS Institute Inc., NC, USA).

## Results

### Subcutaneous administration of WJ-MSCs ameliorates *Af*-induced AD in a murine model

**Fig. 1:**
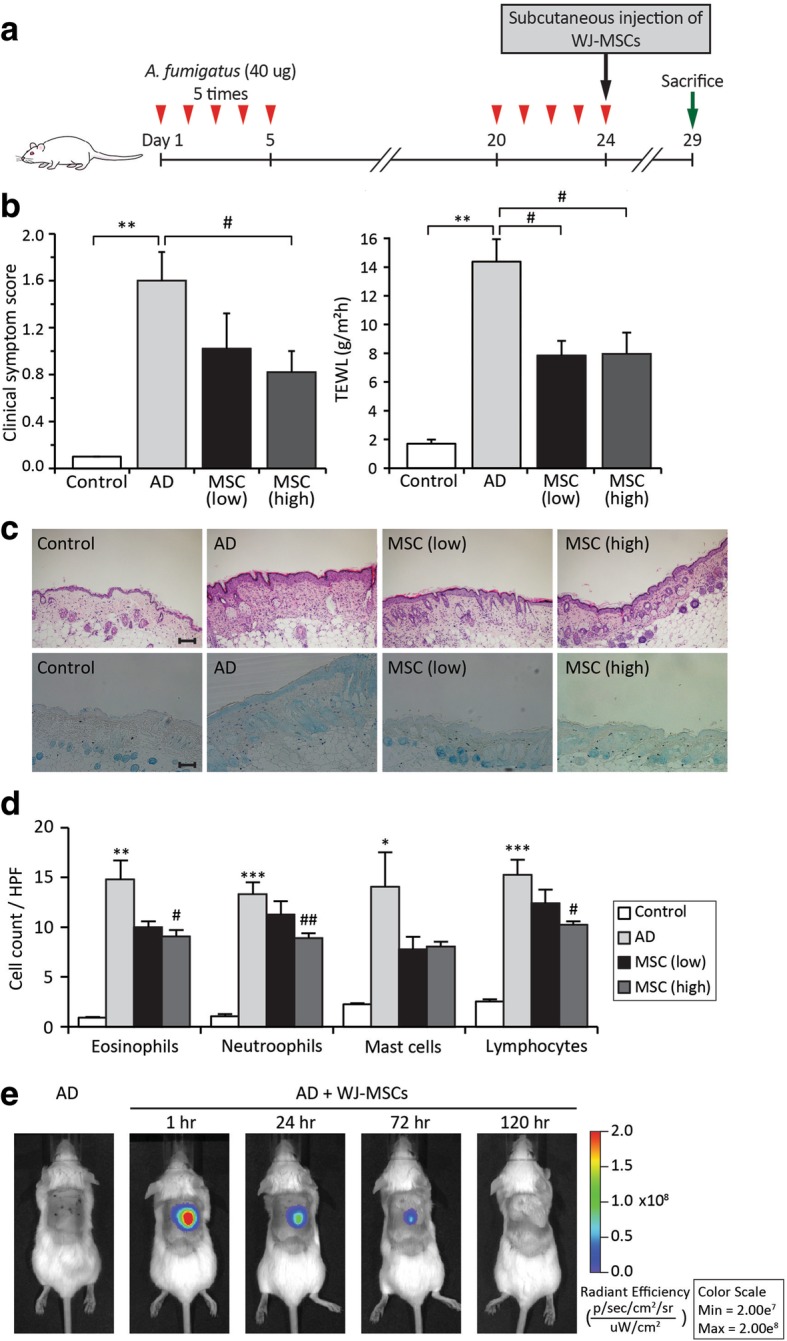
Wharton’s jelly-derived mesenchymal stem cells (WJ-MSCs) improve *Aspergillus fumigatus* (*Af*)-induced atopic dermatitis (AD) in a murine model. **a** Study design. A murine model of AD was developed by applying *Af* extract to the dorsal skin of BALB/c mice for five consecutive days and then repeating this application after 2 weeks. WJ-MSCs were subcutaneously injected on day 24 and the effects were examined on day 29. **b** Clinical symptom scores (left) and transepidermal water loss (TEWL, right) were determined after injection of high and low doses of WJ-MSCs. **c** Histopathologic features of skin lesions. (Top) Skin lesions were stained with haematoxylin-eosin to identify eosinophils, neutrophils and lymphocytes. (Bottom) Skin lesions were stained with toluidine blue to identify mast cells. Scale bar = 100 μm. **d** Numbers of eosinophils, neutrophils, mast cells and lymphocytes were counted under a microscope at × 200 magnification. Data are presented as the mean ± SEM (*N* = 6 per group). **, *p* < 0.005; ***, *p* < 0.0005 compared with the control group; #, *p* < 0.05; ##, *p* < 0.005 compared with the AD group. **e** Qtracker® 800-labelled WJ-MSCs were traced in vivo using the IVIS Spectrum in vivo imaging system. Fluorescent signals were strongest at the injection site after 1 h and persisted for up to 72 h

We evaluated the therapeutic effects of WJ-MSCs in mice with *Af*-induced AD. *Af* is one of the most commonly encountered species of mould in daily life [14]. *Af* extract (40 μg) was applied to the dorsal skin of BALB/c mice twice with an interval of 2 weeks (Fig. 1a). Mice were subcutaneously injected with WJ-MSCs on day 24, the last day of *Af* extract application, and the effects were examined on day 29. We first compared the therapeutic effects of low (2 × 10^6^) and high (4 × 10^6^) doses of WJ-MSCs to determine the optimal cell dose. Clinical symptom scores and TEWL were significantly lower in mice administered low and high doses of WJ-MSCs than in mice not administered WJ-MSCs (Fig. 1b). In addition, histopathologic examination revealed that dermal inflammation and epidermal hyperplasia were reduced in mice administered low and high doses of WJ-MSCs (Fig. 1c). Furthermore, the numbers of immune cells, including eosinophils, neutrophils, mast cells and lymphocytes, were much lower in mice administered low and high doses of WJ-MSCs than in mice not administered WJ-MSCs (Fig. 1d and Additional file 1). Overall, there were no significant differences between mice administered low and high doses of WJ-MSCs. Therefore, mice were administered a low dose of WJ-MSCs in all subsequent experiments.

To monitor the in vivo distribution of a low dose of WJ-MSCs in the murine model of AD, we subcutaneously injected mice with Qtracker® 800-labelled WJ-MSCs and traced these cells using the IVIS Spectrum imaging system (Fig. 1e). Fluorescent signals were strongest at the injection site after 1 h and persisted for up to 72 h.

### Priming with pro-inflammatory molecules affects the transcriptome profiles and key regulatory pathways in WJ-MSCs

**Fig. 2:**
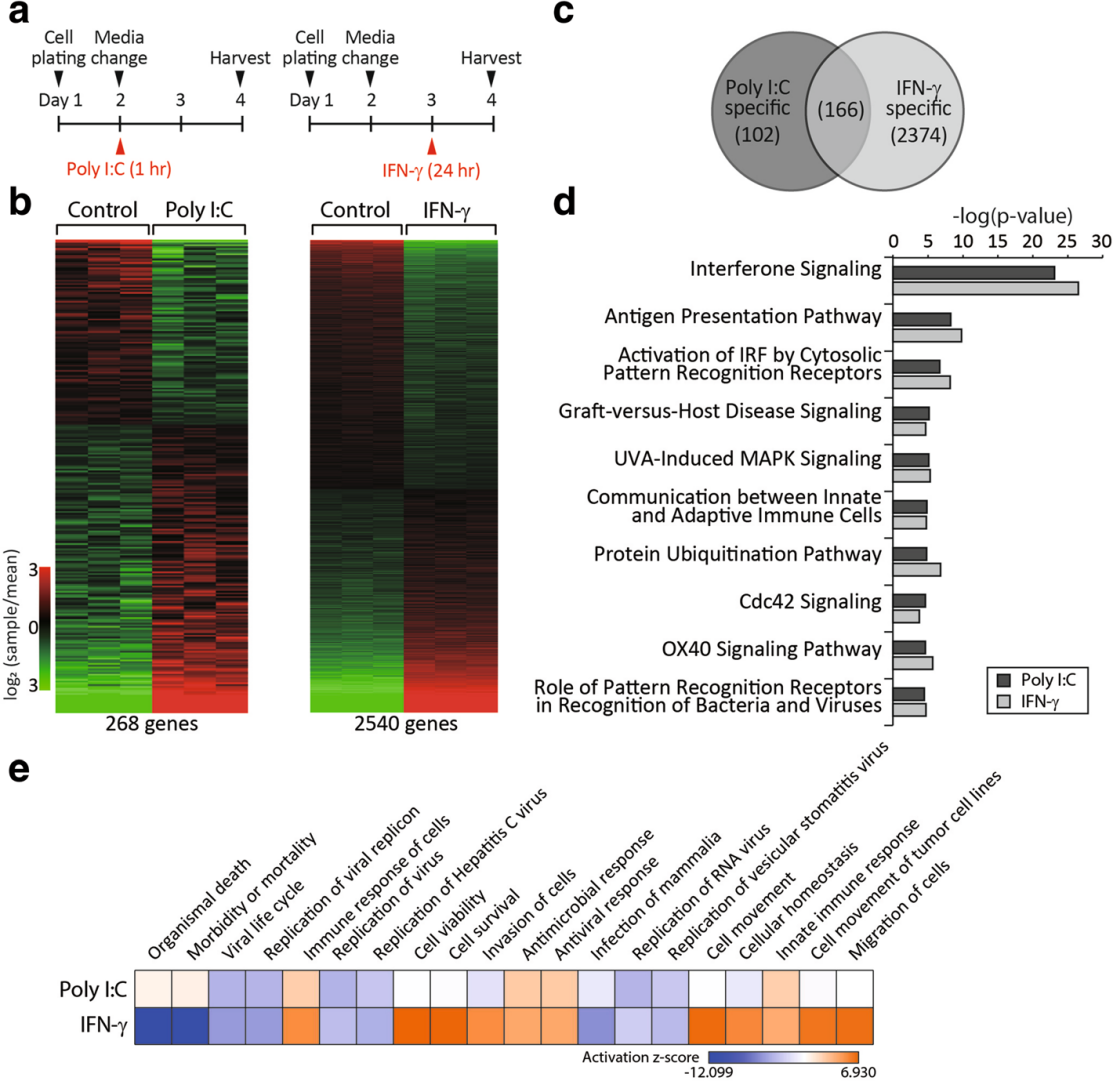
Priming with pro-inflammatory molecules affects the gene expression profiles of Wharton’s jelly-derived mesenchymal stem cells (WJ-MSCs). **a** Schematics of the priming of WJ-MSCs with poly I:C or interferon-gamma (IFN-γ). **b** Heat maps of upregulated (red) and downregulated (green) genes in poly I:C- and IFN-γ-primed WJ-MSCs compared with non-primed WJ-MSCs (control). **c** Venn diagram of mRNAs commonly or differentially expressed in poly I:C- and IFN-γ-primed WJ-MSCs. **d** Top 10 canonical pathways commonly enriched in poly I:C- and IFN-γ-primed WJ-MSCs. **e** A heat map of the top 20 diseases and bio-functions enriched in poly I:C- or IFN-γ-primed WJ-MSCs. Diseases and bio-functions are sorted by the activation *z*-score

We confirmed that WJ-MSCs elicited immunosuppressive effects in mice with *Af*-induced AD (Fig. 1), consistent with previous studies in which different AD models were treated with MSCs derived from sources other than Wharton’s jelly [15]. However, the functions of non-primed MSCs are limited, and great efforts have been recently made to enhance the therapeutic effects of MSCs in various immunological disorders [16–19]. We primed WJ-MSCs with one of two pro-inflammatory molecules, the TLR3 agonist poly I:C and IFN-γ, to enhance their immunomodulatory functions. We established the priming schemes based on previous reports to maximise the immunosuppressive effects of WJ-MSCs [11, 20]. Specifically, WJ-MSCs were treated with poly I:C for 1 h and with IFN-γ for 24 h (Fig. 2a). We compared the global mRNA profiles of non-primed and primed WJ-MSCs to elucidate how these pro-inflammatory molecules affect stem cells at the molecular level. Among 47,231 probes tested using the Human HT-12 v4.0 Expression BeadChip, expression of 268 transcripts differed between poly I:C-primed and non-primed WJ-MSCs (Fig. 2b, left, Student’s *t* test, *p* < 0.001). In addition, expression of 2540 transcripts differed between IFN-γ-primed and non-primed WJ-MSCs (Fig. 2b, right, Student’s *t* test, *p* < 0.001). Among these, 166 transcripts were commonly expressed in both poly I:C- and IFN-γ-primed WJ-MSCs. By contrast, 102 and 2374 transcripts were expressed specifically in poly I:C- and IFN-γ-primed WJ-MSCs, respectively (Fig. 2c). We performed pathway analysis of all differentially expressed transcripts in each condition using IPA software (http://www.ingenuity.com/). Canonical pathways involved in interferon signalling, the antigen presentation pathway and communication between innate and adaptive immune cells were commonly enriched in both poly I:C- and IFN-γ-primed WJ-MSCs (Fig. 2d). Next, we performed comparative function and network analyses of the 102 and 2374 transcripts that were specifically expressed in poly I:C- and IFN-γ-primed WJ-MSCs, respectively. Organismal death and morbidity/mortality functions were enriched in poly I:C-primed WJ-MSCs (Fig. 2e, top column, activation z-score of − 4.036 to 2.611), whereas immune response of cells, cell viability/survival and cell movement functions were enriched in IFN-γ-primed WJ-MSCs (Fig. 2e, bottom column, activation *z*-score of − 12.099 to 6.930).

### Priming with poly I:C or IFN-γ improves the therapeutic effects of WJ-MSCs in AD mice

**Fig. 3:**
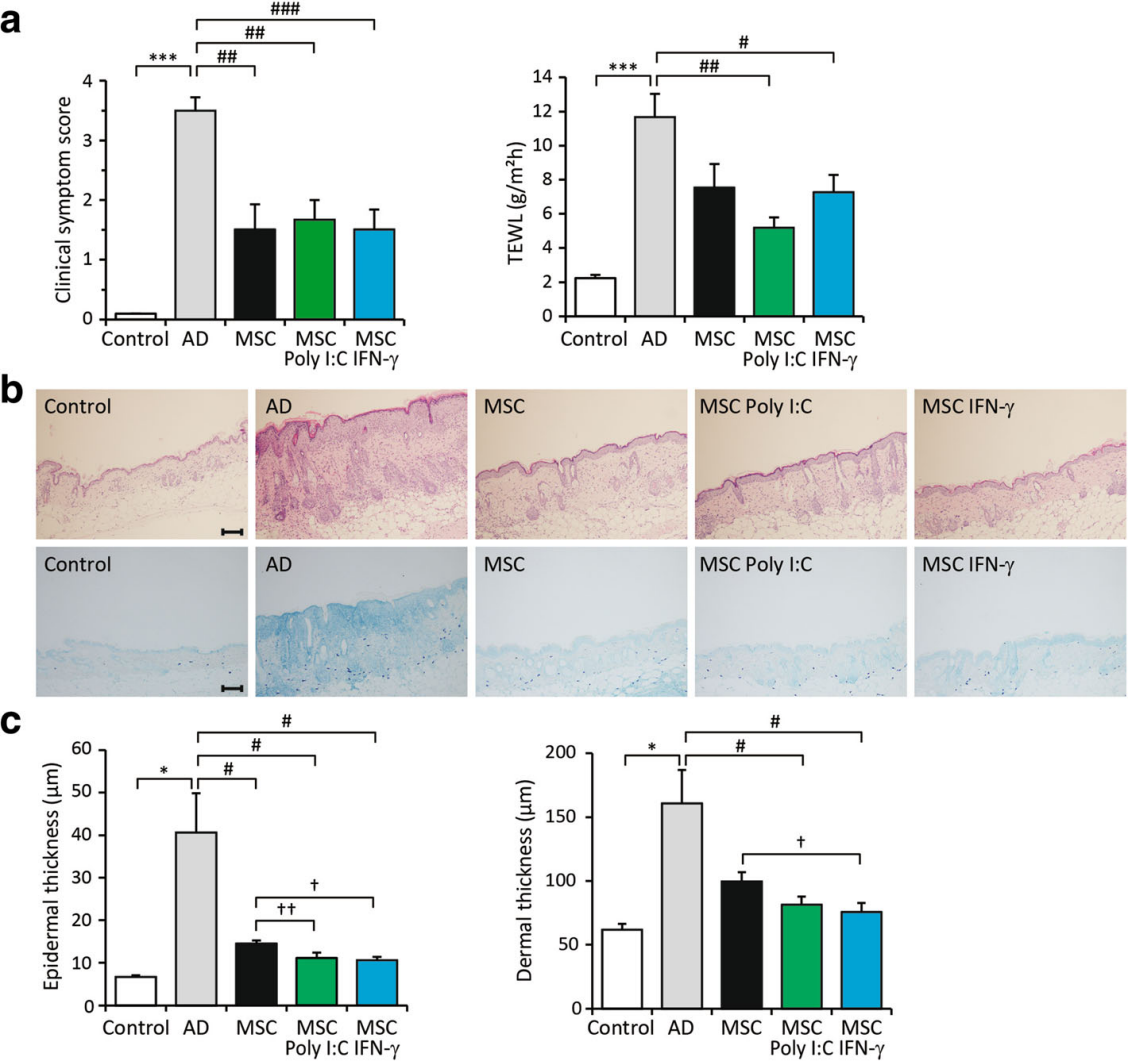
Primed Wharton’s jelly-derived mesenchymal stem cells (WJ-MSCs) elicit enhanced therapeutic effects in mice with *Aspergillus fumigatus* (*Af*)-induced atopic dermatitis (AD). **a** Mice were subcutaneously injected with WJ-MSCs primed with poly I:C or interferon-gamma (IFN-γ) on the final day of *Af* application and sacrificed on day 29 for further analyses. Clinical symptom scores (left) and transepidermal water loss (TEWL) were determined. **b** Histopathologic features of skin. Staining with haematoxylin-eosin (top) and toluidine blue (bottom). **c** Epidermal and dermal thickness was measured. Data are presented as the mean ± SEM (*N* = 6 per group). **, *p* < 0.005; ***, *p* < 0.0005 compared with the control group; #, *p* < 0.05 compared with the AD group; †, *p* < 0.05; ††, *p* < 0.005 compared with the group injected with non-primed WJ-MSCs. Scale bar = 100 μm

Microarray analysis revealed that priming with poly I:C or IFN-γ altered the immunomodulatory functions of WJ-MSCs. We therefore next examined the effects of primed WJ-MSCs in AD mice. WJ-MSCs were primed with poly I:C or IFN-γ and then subcutaneously injected into mice on day 24, as shown in Fig. 1. Non-primed, poly I:C-primed, and IFN-γ-primed WJ-MSCs all reduced clinical symptom scores to a similar extent (Fig. 3a, left). By contrast, TEWL was reduced most in mice administered poly I:C-primed WJ-MSCs (mice administered non-primed WJ-MSCs, 7.5 ± 1.4 g/m^2^h vs. mice administered poly I:C-primed WJ-MSCs, 5.2 ± 1.4 g/m^2^h, *p* = 0.152) (Fig. 3a, right, green bar). Examination of histopathologic features revealed that epidermal thickness was significantly lower in mice administered primed WJ-MSCs than in mice administered non-primed WJ-MSCs (Fig. 3b). Epidermal thickness was lower in mice administered poly I:C-primed (11.1 ± 3.1 μm) and IFN-γ-primed (10.5 ± 2.1 μm) WJ-MSCs than in mice administered non-primed WJ-MSCs (14.4 ± 1.9 μm) (Fig. 3c). Dermal thickness was significantly lower in mice administered IFN-γ-primed WJ-MSCs (75.7 ± 16.9 μm) than in mice administered non-primed WJ-MSCs (99.7 ± 17.7 μm) (*p* < 0.05) (Fig. 3c, right). However, dermal thickness was not significantly decreased in mice administered poly I:C-primed WJ-MSCs.

### Administration of poly I:C- or IFN-γ-primed WJ-MSCs significantly decreases the numbers of various types of immune cells in skin lesions of AD mice

**Fig. 4:**
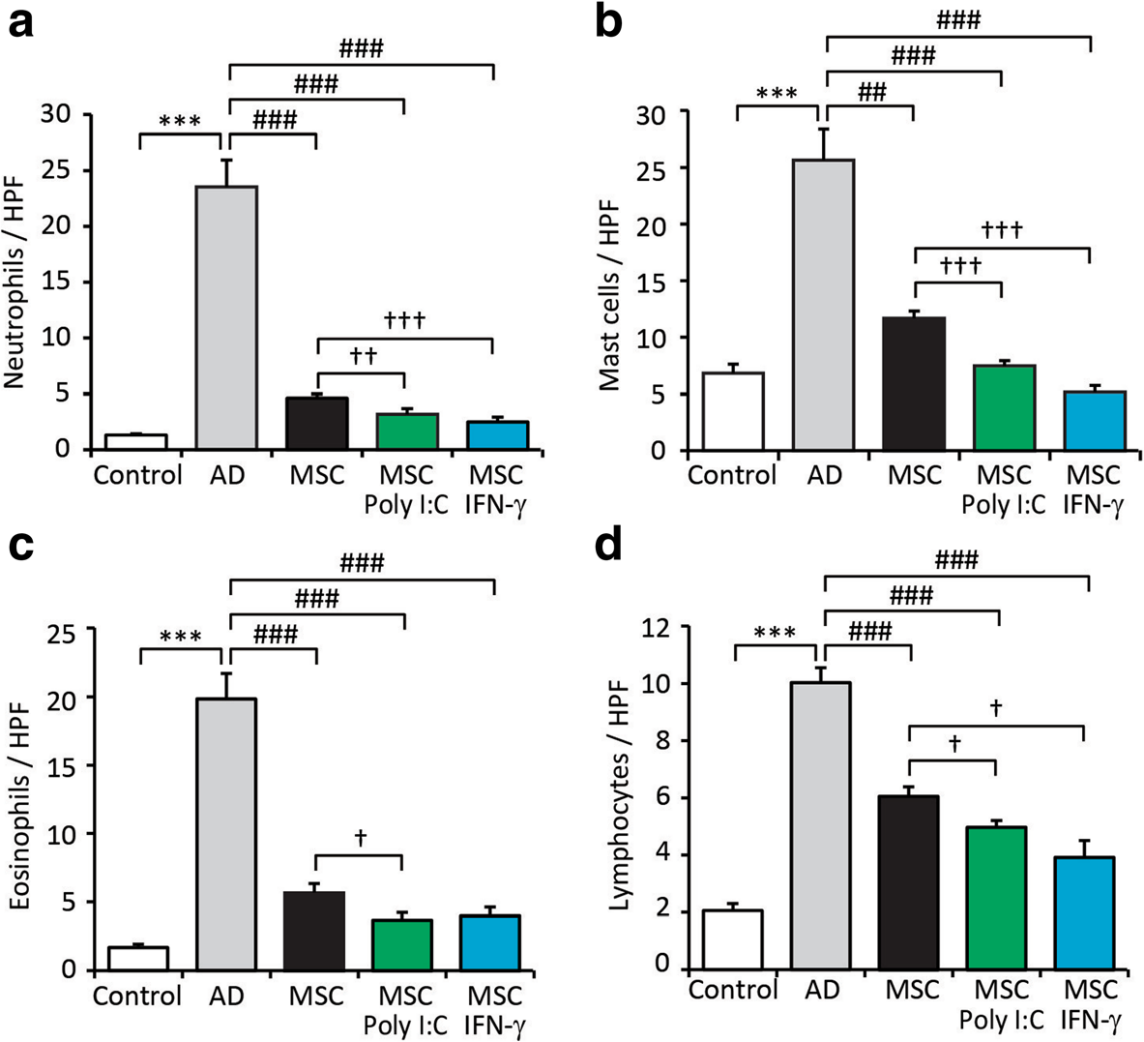
Administration of primed Wharton’s jelly-derived mesenchymal stem cells (WJ-MSCs) significantly decreases immune cell infiltration in mice with *Aspergillus fumigatus* (*Af*)-induced atopic dermatitis (AD). Immune cells were examined at lesion and/or injected sites. Numbers of **a** neutrophils, **b** mast cells, **c** eosinophils and **d** lymphocytes were counted under a microscope. Data are presented as the mean ± SEM (*N* = 6 per group). ***, *p* < 0.0005 compared with the control group; #, *p* < 0.05; ##, *p* < 0.005; ###, *p* < 0.0005 compared with the AD group; †, *p* < 0.05; ††, *p* < 0.005; †††, *p* < 0.0005 compared with the group injected with non-primed WJ-MSCs. HPF, high-power field

To investigate the local inflammatory response after administration of primed WJ-MSCs, we examined various types of immune cells at lesion/injection sites. The numbers of major effector cells in allergic reactions, including neutrophils, mast cells, eosinophils and lymphocytes, were significantly lower in mice administered poly I:C-primed WJ-MSCs than in mice administered non-primed WJ-MSCs (Fig. 4a–d and Additional file 2). The numbers of neutrophils, mast cells and lymphocytes were significantly lower in mice administered IFN-γ-primed WJ-MSCs than in mice administered non-primed WJ-MSCs (Fig. 4a, b, d and Additional file 2).

### Administration of primed WJ-MSCs affects immune responses in LNs of AD mice

**Fig. 5:**
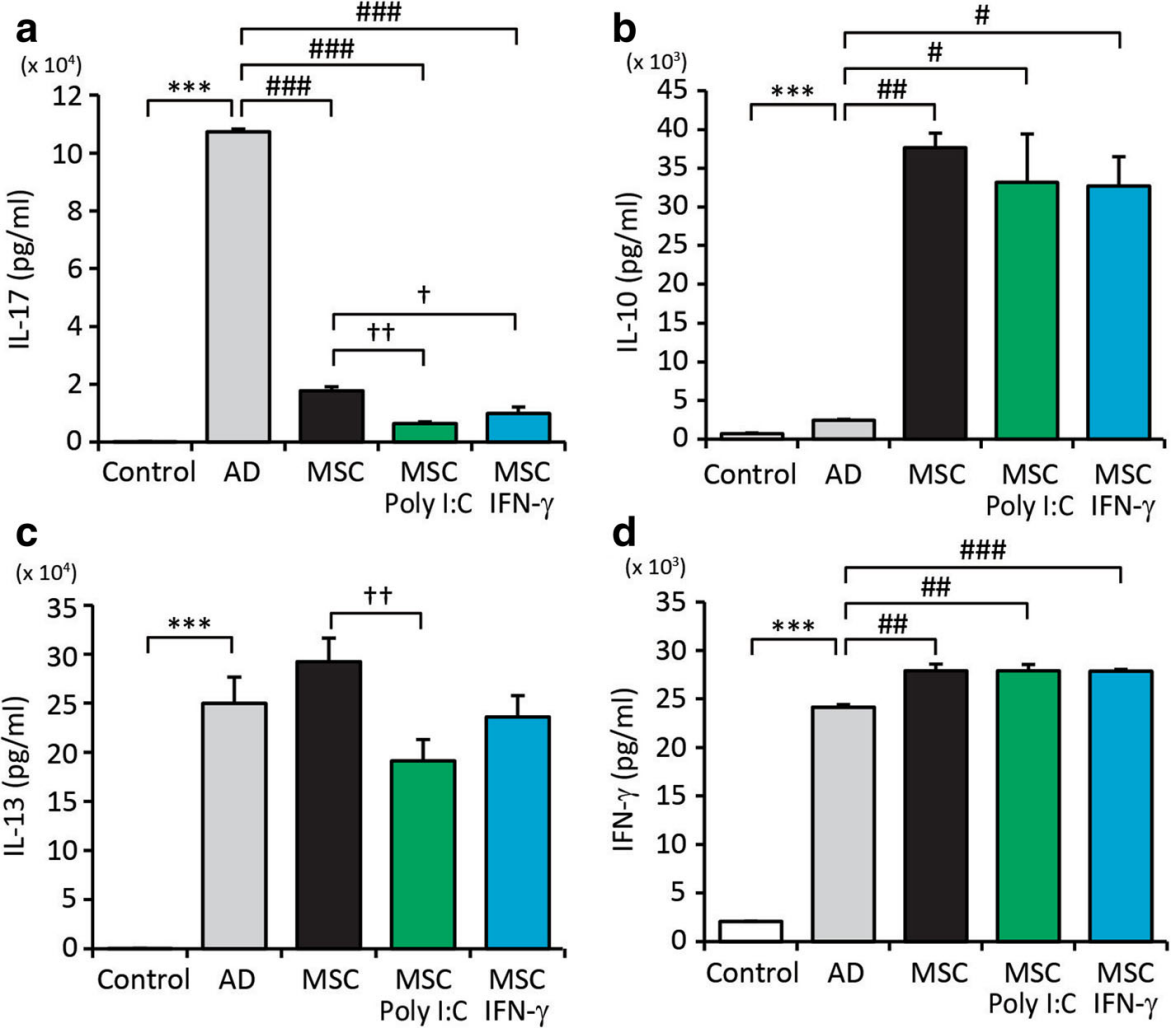
Administration of primed Wharton’s jelly-derived mesenchymal stem cells (WJ-MSCs) significantly changes cytokine levels in lymph node (LN) cells. Skin draining LN cells were stimulated with anti-CD3/CD28 antibodies for 2 days. Levels of **a** interleukin (IL)-17, **b** IL-10, **c** IL-13 and **d** interferon-gamma (IFN-γ) in the culture medium were measured by enzyme-linked immunosorbent assays (ELISAs). Data are presented as the mean ± SEM (*N* = 6 per group). **, *p* < 0.005; ***, *p* < 0.0005 compared with the control group; #, *p* < 0.05 compared with the AD group

We next investigated the global consequences of primed WJ-MSCs on *Af*-induced AD. To this end, we stimulated skin draining LN cells obtained from mice with anti-CD3/CD28 antibodies in vitro, collected the culture medium and measured the levels of various immunomodulatory cytokines, including IL-10, IL-13, IL-17 and IFN-γ, using ELISAs. Overall, cytokine concentrations markedly differed between the culture medium of LN cells derived from mice administered non-primed or primed WJ-MSCs and the culture medium of LN cells derived from non-injected mice, suggesting that WJ-MSCs elicited therapeutic effects (Fig. 5). However, the concentrations of specific cytokines significantly differed between the culture medium of LN cells derived from mice administered non-primed WJ-MSCs and the culture medium of LN cells derived from mice administered primed WJ-MSCs. The changes were most pronounced in the culture medium of LN cells derived from mice administered poly I:C-primed WJ-MSCs. For example, the concentration of IL-17 was 64.2% lower in the culture medium of LN cells derived from mice administered poly I:C-primed WJ-MSCs than in the culture medium of LN cells derived from mice administered non-primed WJ-MSCs (6.38 ng/mL vs. 17.81 ng/mL, *p* < 0.005). In addition, the concentration of IL-13 was 34.6% lower in the culture medium of LN cells derived from mice administered poly I:C-primed WJ-MSCs than in the culture medium of LN cells derived from mice administered non-primed WJ-MSCs (191.08 ng/mL vs. 292.24 ng/mL, *p* < 0.005). By contrast, the concentration of IL-17 was 43.9% lower in the culture medium of LN cells derived from mice administered IFN-γ-primed WJ-MSCs than in the culture medium of LN cells derived from mice administered non-primed WJ-MSCs (9.98 ng/mL vs. 17.81 ng/mL, *p* < 0.05). Taken together, these results suggest that poly I:C-primed MSCs elicit improved therapeutic effects in AD mice partly by decreasing secretion of IL-13 and/or IL-17.

### Distinct molecular and cellular functions underlie the enhanced therapeutic effects of primed WJ-MSCs in AD mice

**Fig. 6:**
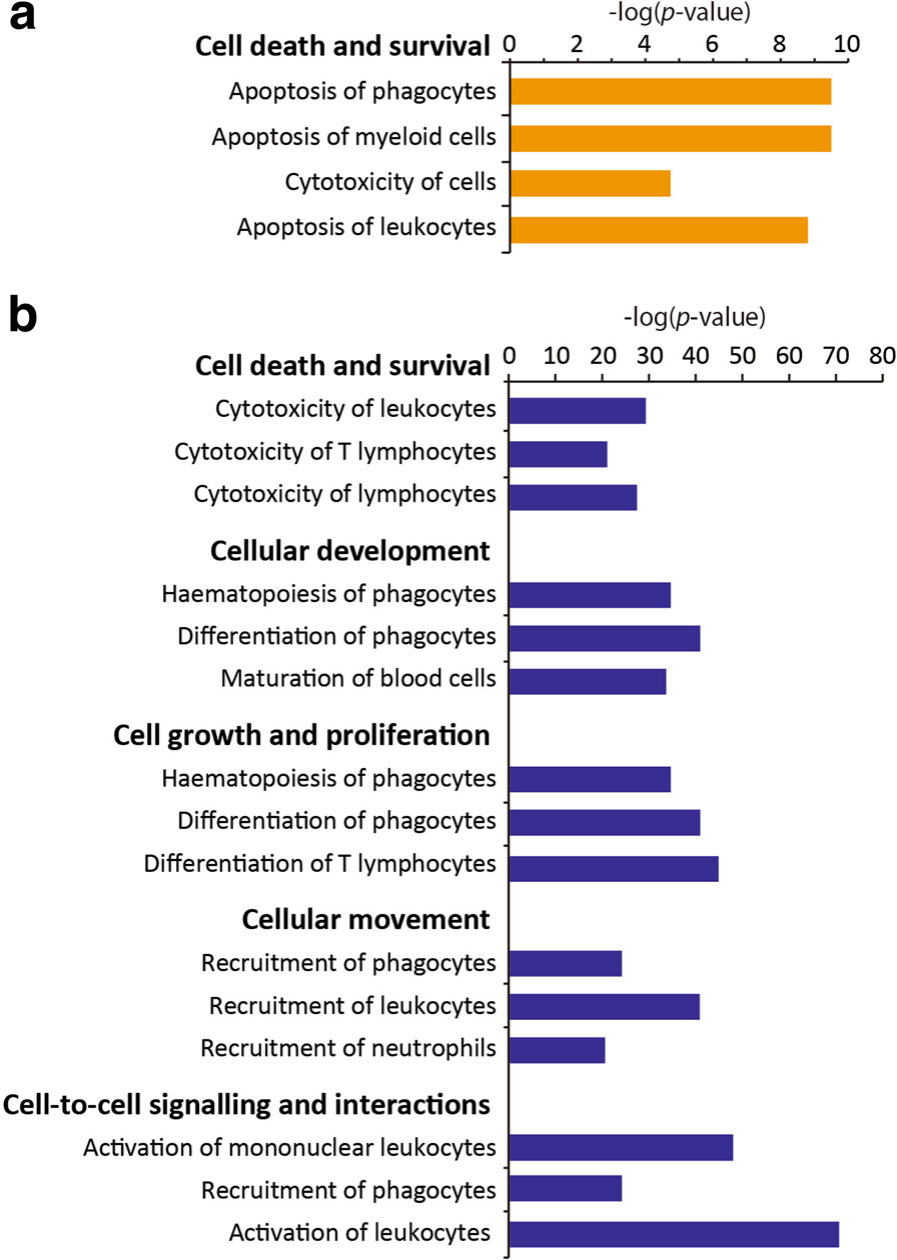
Molecular and cellular functions associated with the upstream regulators expressed in primed Wharton’s jelly-derived mesenchymal stem cells (WJ-MSCs). Molecular and cellular functions associated with the upstream regulators expressed in **a** poly I:C-primed WJ-MSCs and **b** interferon-gamma (IFN-γ)-primed WJ-MSCs

To investigate the mechanisms underlying the enhanced therapeutic effects of primed WJ-MSCs, we performed extensive bioinformatics analyses. We hypothesised that upstream regulators expressed in primed WJ-MSCs underlie the aforementioned changes in cytokine secretion in LNs of AD mice. Using IPA software, we first identified potential upstream regulators of the cytokines IL-17, IL-10, IL-13 and IFN-γ. A total of 1697 molecules, including genes and small molecules, were predicted to be upstream regulators. Among these, 25 and 198 regulator genes were expressed in poly I:C- and IFN-γ-primed WJ-MSCs, respectively (Additional files 3 and 4). The expressed upstream regulators were then functionally analysed by IPA using an activation *z*-score of ≥ 2 as a cut-off, which is considered significant [21]. Surprisingly, the upstream regulators expressed in poly I:C- and IFN-γ-primed WJ-MSCs were enriched in distinct functions. The 25 upstream regulators expressed in poly I:C-primed WJ-MSCs were predominantly involved in cell death and survival, including apoptosis of various types of immune cells (Fig. 6a and Additional file 5). By contrast, the 198 upstream regulators expressed in IFN-γ-primed WJ-MSCs were involved in diseases and other functions, in addition to cell death and survival. For instance, upstream regulators of cellular development, cell growth and proliferation, cell movement, cell-to-cell signalling and interactions and cell-mediated immune responses were significantly activated (Fig. 6b and Additional file 6).

## Discussion

MSCs elicit immunomodulatory effects and are a promising therapeutic candidate for allergic diseases, such as asthma, allergic rhinitis and AD [13, 15, 17–19]. Recent studies focused on developing new strategies to maximise the therapeutic effects of MSCs, including improving their survival and/or modulating their immunomodulatory responses via preconditioning. Cytokine treatment, hypoxic culture and three-dimensional culture affect the functions of MSCs [8, 11, 16, 22, 23]. Here, we assessed whether priming with the TLR3 agonist poly I:C or IFN-γ enhances the therapeutic effects of WJ-MSCs in a murine model of AD.

TLRs participate in the immune response during infections by inducing expression of inflammatory cytokines and upregulating co-stimulatory molecules, which can modulate the immunoregulatory functions of MSCs [10, 11]. In addition, IFN-γ is a well-known inducer of immune responses, and IFN-γ treatment enhances the immunosuppressive effects of MSCs in vitro and in vivo [20, 24, 25]. Analysis of global mRNA profiles revealed that canonical pathways associated with cell survival and inflammatory responses, including interferon signalling and the antigen presenting pathway, were commonly enriched in WJ-MSCs primed with poly I:C or IFN-γ in comparison with non-primed WJ-MSCs. These results suggest that stimulation with poly I:C or IFN-γ affects the immunomodulatory functions of WJ-MSCs. Consistently, WJ-MSCs primed with poly I:C or IFN-γ elicited improved therapeutic effects in mice with *Af*-induced AD.

Administration of poly I:C- or IFN-γ-primed WJ-MSCs improved the features of AD and reduced clinical symptom scores, TEWL and epidermal thickness in AD-like skin lesions. In addition, administration of primed WJ-MSCs affected infiltration of immune cells into skin lesions and secretion of cytokines in LNs. Specifically, administration of poly I:C-primed WJ-MSCs reduced infiltration of mast cells, lymphocytes, neutrophils and eosinophils, while administration of IFN-γ-primed WJ-MSCs reduced infiltration of neutrophils, mast cells and lymphocytes. Administration of poly I:C-primed WJ-MSCs reduced secretion of IL-13 and IL-17, while administration of IFN-γ-primed WJ-MSCs only reduced secretion of IL-17. Taken together, these results indicate that poly I:C- and IFN-γ-primed WJ-MSCs modulate *Af*-induced immune responses via different mechanisms; poly I:C-primed WJ-MSCs control both eosinophil-associated Th2 immunity and neutrophil-related Th17 immunity, while IFN-γ-primed WJ-MSCs mostly regulate Th17 immune responses.

Th2 cell-mediated responses are dominant in the acute phase of AD [4]. Th17 cell-mediated responses are also involved in the development of AD and contribute to the shift toward the late phase of this disease [26, 27]. In this study, administration of poly I:C- or IFN-γ-primed WJ-MSCs suppressed Th17 immune responses in LNs. These results suggest that poly I:C- or IFN-γ-primed MSCs can be used for the clinical treatment of AD in part by modulation of Th17 immunity.

MSCs elicit immunomodulatory effects by suppressing T cell proliferation, regulating Tregs and producing co-stimulatory molecules of B cells [28–31]. However, systematic analysis of underlying mechanisms other than immune cell regulation has not been extensively studied. To elucidate the molecular mechanisms underlying the immunomodulatory effects of primed WJ-MSCs, we performed comprehensive transcriptome profiling and pathway/network analyses. Global mRNA profiles revealed that canonical pathways involved in interferon signalling, the antigen presentation pathway and communication between innate and adaptive immune cells were commonly enriched in WJ-MSCs primed with poly I:C or IFN-γ. However, distinct regulatory pathways were also specifically enriched in poly I:C- or IFN-γ-primed WJ-MSCs. For example, organismal death and morbidity/mortality functions were enriched in poly I:C-primed WJ-MSCs, whereas immune responses of cells, cell survival and cell movement functions were enriched in IFN-γ-primed WJ-MSCs. Next, we investigated the effect of primed WJ-MSCs on cytokine secretion in LNs. We first identified possible upstream regulators of IL-17 and IL-13, whose secretion in LNs was affected by injection of WJ-MSCs. Among more than 1000 predicted upstream regulators of these cytokines, 25 and 198 were expressed in poly I:C- and IFN-γ-primed WJ-MSCs, respectively. Using these datasets, we performed comprehensive pathway and network analyses. Surprisingly, pathways and networks associated with distinct molecular and cellular functions were enriched in poly I:C- or IFN-γ-primed WJ-MSCs, which may underlie the differences in their modes of action. For example, priming with poly I:C considerably affected cell death and survival functions, including apoptosis of immune cells, whereas priming with IFN-γ affected much more complex functions, including cellular development, cell growth and proliferation, cell-to-cell signalling and interactions, cell-mediated immune responses and cell movement. These data suggest that primed WJ-MSCs affected diverse cellular responses, such as apoptosis, cell proliferation and cell movement, in addition to well-known T cell functions. Clearly, there are overlapping and distinctive pathways associated with each priming condition. Consistently, poly I:C- and IFN-γ-primed WJ-MSCs had similar, but subtly different, therapeutic effects. Therefore, priming of stem cells should be optimised to target specific pathways, depending on the pathophysiological context.

Taken together, this study demonstrates that priming with poly I:C or IFN-γ enhances the therapeutic effects of WJ-MSCs on AD partly by modulating distinct regulatory pathways and/or networks.

## Conclusions

Priming with poly I:C or IFN-γ affected the immunomodulatory functions of WJ-MSCs and enhanced their therapeutic effects on AD. Administration of poly I:C- or IFN-γ-primed WJ-MSCs alleviated the features of AD including the clinical symptom score, TEWL and epidermal thickness. Priming of WJ-MSCs regulated immune responses via enrichment of canonical pathways involved in interferon signalling, the antigen presentation pathway and communication between innate and adaptive immune cells. Moreover, each priming showed discrete enrichment of molecular and cellular functions, which may explain their different mechanisms of action. In addition to commonly enriched pathways, priming with poly I:C considerably affected cell death and survival functions, including apoptosis of immune cells, whereas priming with IFN-γ affected much more complex functions, including cellular development, cell growth and proliferation, cell-to-cell signalling and interactions, cell-mediated immune responses and cell movement. IL-13 and IL-17 levels were reduced in the culture medium of LN cells derived from mice administered poly I:C-primed WJ-MSCs, while only the IL-17 level was reduced in the culture medium of LN cells derived from mice administered IFN-γ-primed WJ-MSCs. Priming of stem cells should be optimised to target specific pathways, depending on the pathophysiological context.

Taken together, this study demonstrates that priming with poly I:C or IFN-γ enhances the therapeutic effects of WJ-MSCs on AD partly by modulating distinct regulatory pathways and/or networks.

## Additional files

Additional file 1: **Figure S1.** Administration of Wharton’s jelly-derived mesenchymal stem cells (WJ-MSCs) improves immune cell infiltration in skin lesions of mice with *Aspergillus fumigatus* (*Af*)-induced atopic dermatitis (AD). (Left) Skin lesions were stained with haematoxylin-eosin to identify eosinophils, neutrophils and lymphocytes. (Right) Skin lesions were stained with toluidine blue to identify mast cells. Arrows indicate infiltrated immune cells in skin lesions. Scale bar = 20 μm. (TIF 6307 kb)

Additional file 2: **Figure S2.** Administration of primed Wharton’s jelly-derived mesenchymal stem cells (WJ-MSCs) significantly decreases the numbers of various types of immune cells in skin lesions of mice with *Aspergillus fumigatus* (*Af*)-induced atopic dermatitis (AD). Staining with haematoxylin-eosin (left) and toluidine blue (right). Arrows indicate infiltrated immune cells in skin lesions. Scale bar = 20 μm. (TIF 7986 kb)

Additional file 3: **Table S1.** Upstream regulators of key cytokines that are expressed in poly I:C-primed Wharton’s jelly-derived mesenchymal stem cells (WJ-MSCs). (XLS 31 kb)

Additional file 4: **Table S2.** Upstream regulators of key cytokines that are expressed in interferon-gamma (IFN-γ)-primed Wharton’s jelly-derived mesenchymal stem cells (WJ-MSCs). (XLS 49 kb)

Additional file 5: **Figure S3.** Analysis of networks of molecular and cellular functions in poly I:C-primed Wharton’s jelly-derived mesenchymal stem cells (WJ-MSCs) using Ingenuity Pathway Analysis (IPA) software. The top row represents upstream regulators expressed in poly I:C-primed WJ-MSCs. The molecule types and relationships are indicated in the box. The orange dashed lines indicate the relationships that lead to activation of downstream functions. (TIF 2680 kb)

Additional file 6: **Table S3.** A full list of principal molecular and cellular functions associated with upstream regulators expressed in interferon-gamma (IFN-γ)-primed Wharton’s jelly-derived mesenchymal stem cells (WJ-MSCs). (XLS 34 kb)

## Abbreviations

AD: Atopic dermatitis
Af: *Aspergillus fumigatus*
CD: Cluster of differentiation
DMEM: Dulbecco’s modified Eagle’s medium
ELISA: Enzyme-linked immunosorbent assay
IFN-γ: Interferon-gamma
IL: Interleukin
IPA: Ingenuity Pathway Analysis
LN: Lymph node
MSC: Mesenchymal stem cell
Th: T helper
TLR: Toll-like receptor
WJ-MSC: Wharton’s jelly-derived mesenchymal stem cell
TEWL: Transepidermal water loss
