# Supplementary figures and images for: Priming with Toll-like receptor 3 agonist or interferon-gamma enhances the therapeutic effects of human mesenchymal stem cells in a murine model of atopic dermatitis
Source: Stem Cell Res Ther. 2019 Feb 22;10:66. doi: 10.1186/s13287-019-1164-6 (PMC6387524; doi:10.1186/s13287-019-1164-6)

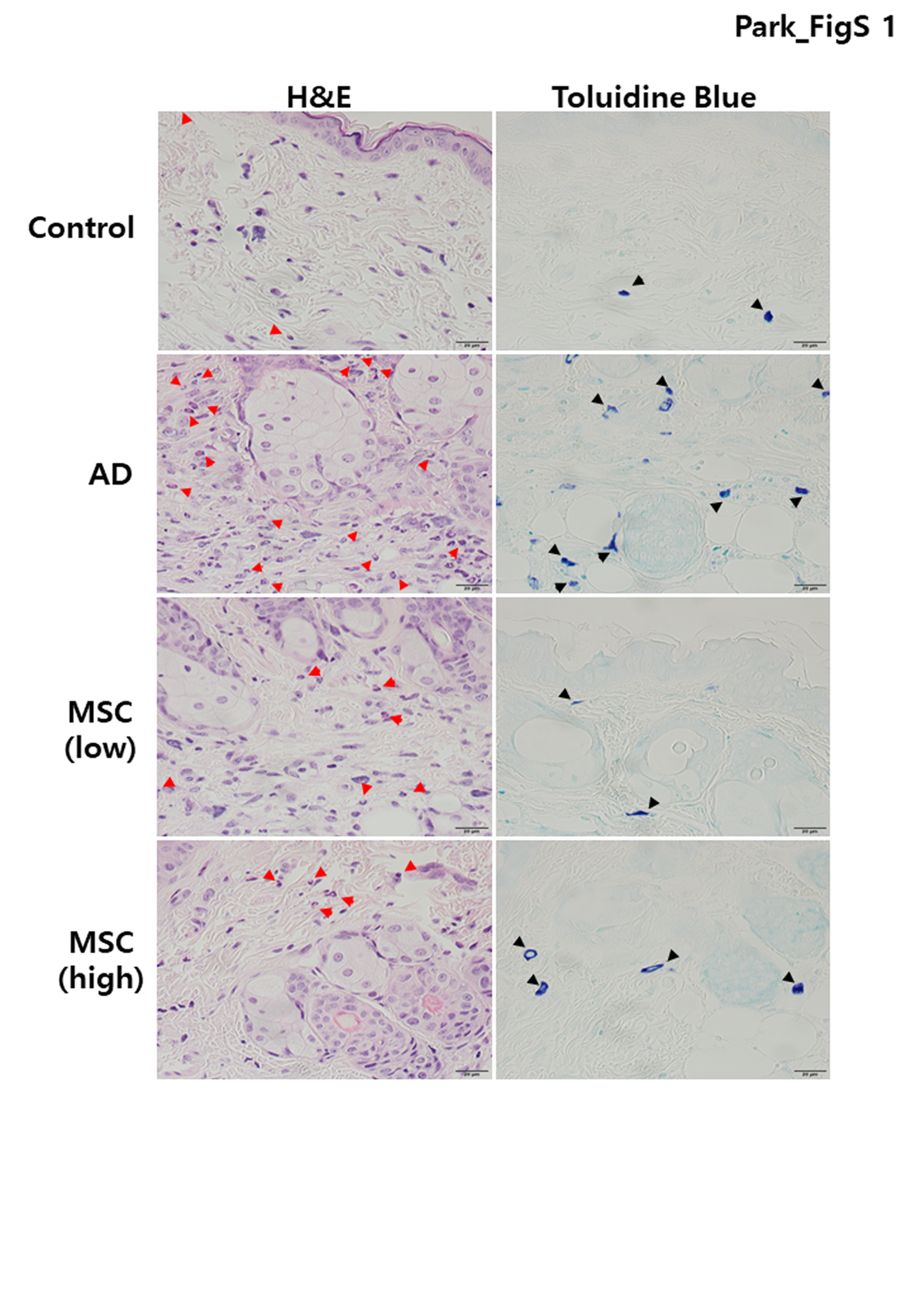

Supplement: Supplementary file 1 — Figure S1. Administration of Wharton’s jelly-derived mesenchymal stem cells (WJ-MSCs) improves immune cell infiltration in skin lesions of mice with Aspergillus fumigatus (Af)-induced atopic dermatitis (AD). (Left) Skin lesions were stained with haematoxylin-eosin to identify eosinophils, neutrophils and lymphocytes. (Right) Skin lesions were stained with toluidine blue to identify mast cells. Arrows indicate infiltrated immune cells in skin lesions. Scale bar = 20 μm. (TIF 6307 kb) [file 13287_2019_1164_MOESM1_ESM.tif]

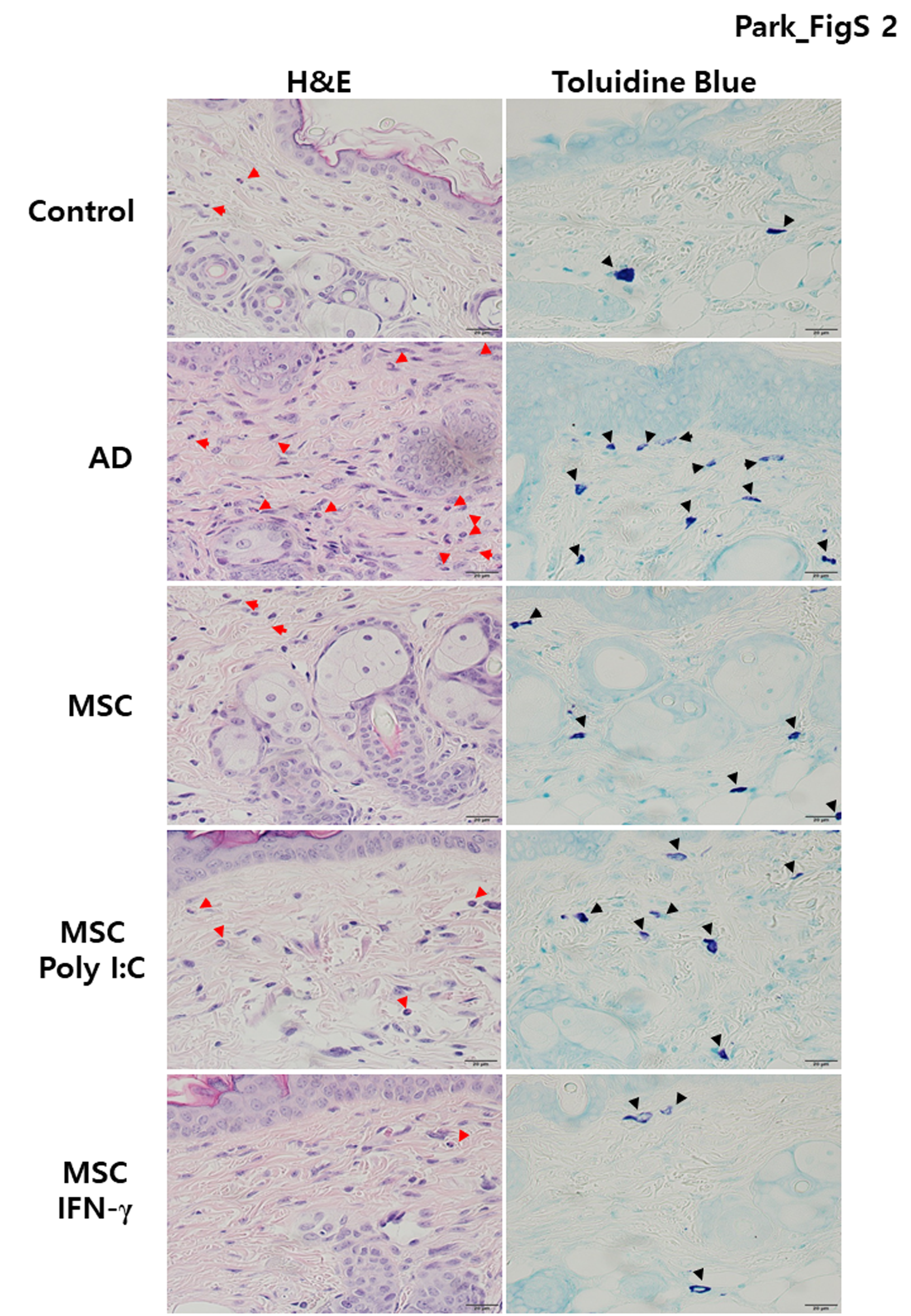

Supplement: Supplementary file 2 — Figure S2. Administration of primed Wharton’s jelly-derived mesenchymal stem cells (WJ-MSCs) significantly decreases the numbers of various types of immune cells in skin lesions of mice with Aspergillus fumigatus (Af)-induced atopic dermatitis (AD). Staining with haematoxylin-eosin (left) and toluidine blue (right). Arrows indicate infiltrated immune cells in skin lesions. Scale bar = 20 μm. (TIF 7986 kb) [file 13287_2019_1164_MOESM2_ESM.tif]

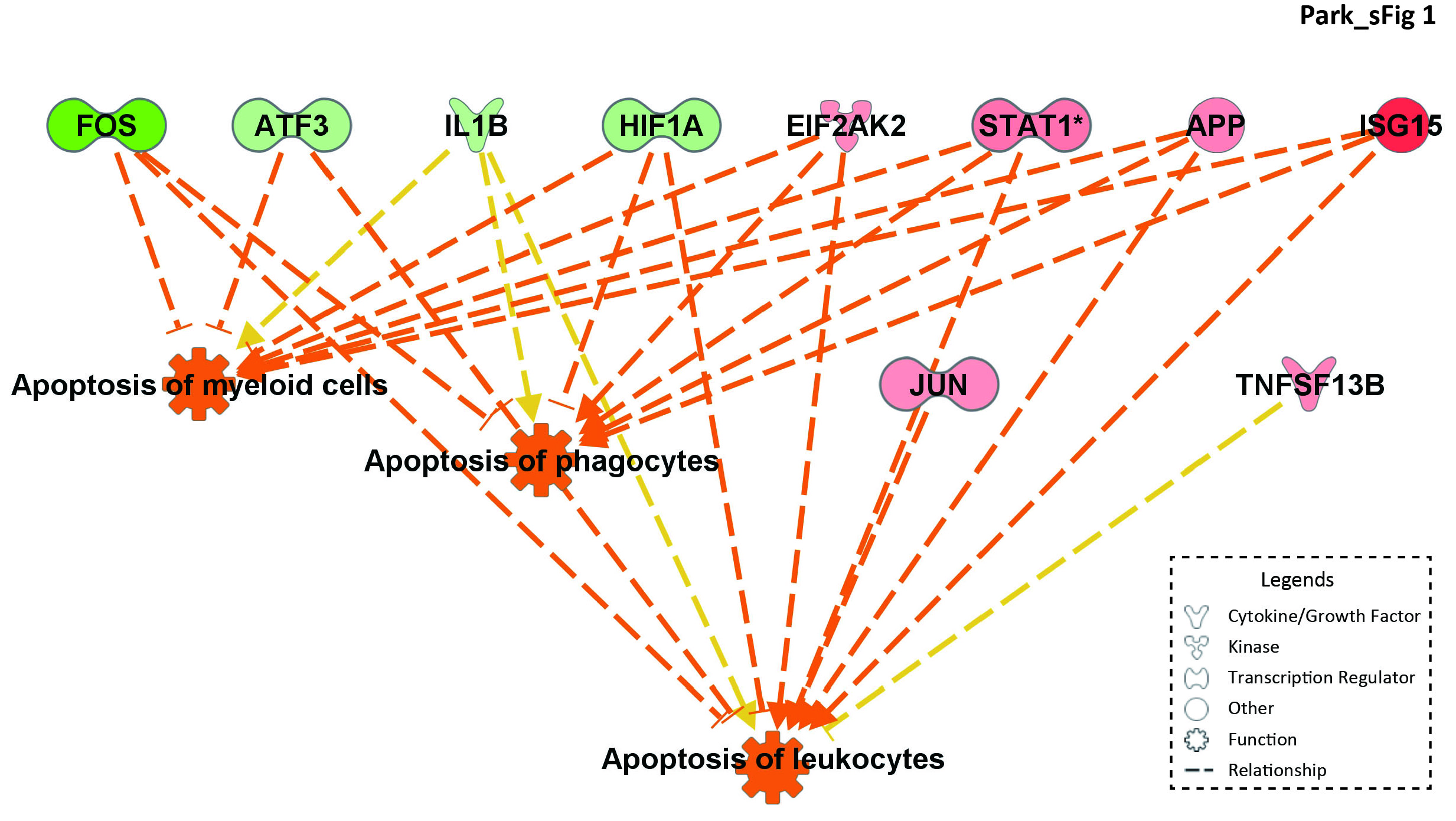

Supplement: Supplementary file 5 — Figure S3. Analysis of networks of molecular and cellular functions in poly I:C-primed Wharton’s jelly-derived mesenchymal stem cells (WJ-MSCs) using Ingenuity Pathway Analysis (IPA) software. The top row represents upstream regulators expressed in poly I:C-primed WJ-MSCs. The molecule types and relationships are indicated in the box. The orange dashed lines indicate the relationships that lead to activation of downstream functions. (TIF 2680 kb) [file 13287_2019_1164_MOESM5_ESM.tif]
